# Climatic factors shaping intraspecific leaf trait variation of a neotropical tree along a rainfall gradient

**DOI:** 10.1371/journal.pone.0208512

**Published:** 2018-12-06

**Authors:** Matheus L. Souza, Alexandre A. Duarte, Maria B. Lovato, Marcilio Fagundes, Fernando Valladares, Jose P. Lemos-Filho

**Affiliations:** 1 Departamento de Botânica, Universidade Federal de Minas Gerais, ICB-UFMG, Belo Horizonte, Brazil; 2 Departamento de Biologia Geral, Universidade Federal de Minas Gerais, ICB-UFMG, Belo Horizonte, Brazil; 3 Departamento de Biologia Geral, Universidade Estadual de Montes Claros, CCBS-UNIMONTES, Montes Claros, Brazil; 4 LINCGlobal Departamento de Biogeografía y Cambio Global, Museo Nacional de Ciencias Naturales, MNCN-CSIC, Madrid, Spain; 5 Departamento de Biología y Geología ESCET, Universidad Rey Juan Carlos, Móstoles, Spain; Estacion Experimental de Zonas Aridas, SPAIN

## Abstract

Intraspecific trait variation has been singled out as an important mechanism by which individuals can cope with environmental variations and avoid local extinctions. Here we evaluate variation in metamer traits (i.e., traits associated with internodes, petioles and their corresponding leaves) and parameters of chlorophyll fluorescence within and among populations of a neotropical tree, *Copaifera langsdorffii*. We also evaluated phenotypic plasticity in natural settings comparing traits between shade and sun-exposed metamers. We selected six populations along a climatic gradient ranging from semi-arid to humid and representing three different biomes (Caatinga, Cerrado, and Atlantic Forest). Local climatic conditions significantly affected the morphological and physiological traits of populations. Trait variation among populations was explained mainly by aridity index and evapotranspiration. Individuals from drier regions had lower specific leaf area (SLA), lower investment in leaf area per total dry mass of metamer (LARm), lower specific petiole length (SPL) and lower potential quantum yield (Fv/Fm, only for sun-exposed metamers). Populations from locations with greater environmental heterogeneity (interannual variation) had greater plasticity in response to light for Fv/Fm and electron transport rate (ETR) and morphological traits related to the hydraulic and biomechanical aspects of the leaves (petiole length, internode length and SPL). High intraspecific variation in metamer traits in *C*. *langsdorffii* coupled with its ability to modify these traits in response to different climate conditions can explain the success of the species over a range of different habitats and represent important factors for the persistence of this species in the face of climate change.

## Introduction

Evaluating the effects of environmental conditions on natural populations is important for understanding the evolutionary processes maintaining biodiversity and the possible impacts that global climate change can have on ecosystems [1–3]. Climate change is expected to increase average global temperature, rainfall variability, and frequency of extreme events, leading to drier environments in many already arid regions [4]. Changes in climate and landscape may influence the availability of resources, which can endanger many species [5]. Due to rapid environmental changes, species may become extinct in large areas of their distributions and only persist in areas more stable climatically, the refugia. They may also: (i) migrate to a more favorable environment, (ii) adjust their functional trait through phenotypic plasticity or (iii) adapt through natural selection [5,6] and these factors may interact synergistically for the survival of the species [7]. However, these responses depend on the intensity and direction of environmental change, life history characteristics, intraspecific genetic variation and interspecific interactions [5,7,8].

Widely distributed species are generally exposed to different environmental pressures and stressful factors such as variation in rainfall, temperature, light and soil fertility, which can vary in intensity and unpredictability [9–12]. For example, widely distributed species across a climate gradient from humid to semi-arid, can have populations subject not only to differential seasonal water limitation but also to differences in interannual precipitation variability [12,13]. The variation in environmental conditions, which widely distributed plant species are exposed is frequently coupled with intraspecific trait variation (ITV) among and within populations[14–19]. On a regional scale, many species have ITV among populations, frequently taking the form of geographic clines that correspond to environmental gradients [14–16,20–22]. Locally, ITV can also be observed among individuals within populations and even within individuals being directed by microenvironmental variations or by unpredictability of the local climate [12,23]. The ITV, among and within populations, is due to different levels of both, genetic variation and phenotypic plasticity [7,24–29].

Phenotypic plasticity is the ability of a genotype to produce different morphological and physiological responses when exposed to different environmental conditions [12,30,31]. Thus, plasticity can dampen the effects of environmental changes that occur throughout the plant life cycle and increase plant tolerance to stress [6,32]. In this sense, phenotypic plasticity is essential for prevention of local extinctions, especially under future climate change scenarios [33,34]. In practice, there are a number of different ways to determine quantitatively phenotypic plasticity through the use of a plethora of plasticity indices [35]. The use of these indices has allowed ecological approaches to phenotypic plasticity, which are relevant in comparative studies of different species and populations [12,36–40]. Distinct from the traditional approach involving common garden or reciprocal transplant experiments [41–43], the degree of phenotypic plasticity of the species or populations can be evaluated directly in natural conditions [39,40,44]. Within a plant, sun-exposed leaves (in outermost portion of the canopy) when compared to shade leaves (in inner part of the canopy) are subjected to different environmental conditions as greater water stress due to high sunlight, higher temperatures and wind action. These different environmental conditions can result in differences in morphological and physiological leaf traits between sun-exposed and shade leaves within an individual due to plasticity phenotypic [45,46]. Sun leaves compared to shade ones are generally smaller, thicker, contain less chlorophyll per unit leaf mass, in addition other changes in leaf biochemical characteristics, which increase carbon gain and water use efficiency [39,47]. In this context, the analysis of phenotypic plasticity in sun-exposed leaves vs. shade leaves provides an excellent system to examine plastic responses to specific environmental cues related to different light conditions or even water stress.

Recent studies have shown that phenotypic plasticity varies positively as a function of environmental heterogeneity, with individuals from populations in heterogeneous environments presenting greater plasticity in functional traits [12,33,48,49]. High phenotypic plasticity in populations of heterogeneous environments can increase their ability to face with climate changes. However, phenotypic plasticity has rarely been considered in the context of the evolutionary responses of plants to climate change along their geographic distributions [12] and can strongly influence the ecological processes related to the growth, survival and reproduction of species in habitats with different environmental filters [11].

Intraspecific trait variation has been singled out as an important mechanism by which individuals can cope with environmental variations, avoid local extinctions in the face of possible climatic changes [33,34]. In spite of this, the influence of specific environmental factors on ITV at different ecological scales is poorly known, mainly in tropical environments (but see [50]). Intraspecific variation in in metamer traits (internode, petiole and corresponding leaf) has been analyzed due to trade-offs observed among these structures and their relationships with differences in environmental conditions [14–16,20–22]. Leaf area and specific leaf area (SLA) are associated with tradeoff between carbon uptake by photosynthesis and water loss by transpiration, which determine resource use efficiency and tolerance to environmental stresses, mainly to water stress [51–53]. Several studies have demonstrated that plants from drier environments have thicker and smaller leaves, lower SLA and lower stomatal conductance [14–16,54]. These characteristics strategy have been largely associated with water use efficiency optimizing plant performance according to environmental conditions with water limitation [12,55]. In addition, other metamer traits, as internode and petiole length and mass are important for the sheet support with regard to the spatial positioning, the biomechanics and hydraulic [56].

The combination of stressful factors and environmental heterogeneity to which widely distributed species are subject makes them excellent models for evaluating the consequences of climate change on natural populations [12,57]. However, studies focusing on ITV levels in neotropical trees are rare [26]. Here, we investigated the effects of climatic variables on morphological and physiological traits of metamers across different ecological scales in *Copaifera langsdorffii*, a widely distributed neotropical tree species along a climatic gradient in southeastern Brazil. We hypothesized that (i) due to climatic gradient and environmental heterogeneity of the sampled area, a high ITV is expected in *C*. *langsdorffii*, (ii) there is a relationship between variation in metamer traits and climatic variables, specific for each trait, and (iii) there is a positive relationship between the degree of phenotypic plasticity and climatic heterogeneity. To testing these hypotheses, we performed partition of ITV in the following hierarchical levels: among populations (regional scale), among individuals within each population (local scale) and within individuals in different light conditions. In order to identify the climatic drivers of the trait variation among populations, we performed multiple regression analyses. The phenotypic plasticity of each trait was estimated by comparing trait values of sun-exposed and shaded metamers within of each individual. We evaluated the effect of climatic heterogeneity (interannual variation) on phenotypic plasticity of populations also with multiple regression analyses.

## Materials and methods

### Species and study area

*Copaifera langsdorffii* Desf (Fabaceae) is a tree species with great variation in size; reproductive adults vary from 2 to 35 m in height, depending on the habitat where they occur [58,59]. This species has a wide distribution in South America [58]. In Brazil, it occurs in four biomes: the Caatinga, Cerrado, Atlantic Forest and Amazon [60]. It presents alternate and compound leaves, with great variation in the number leaflets, which are alternate or opposite and glabrous [60,61]. It has a marked leaf fall during the driest months [62], but the duration of this phenological event varies among populations (unpublished data). Reproduction is supra-annual with seed dispersion in the dry season [62]. *C*. *langsdorffii* seeds are dispersed mainly by animals, particularly birds [63], however seeds not dispersed by birds fall on the forest floor and can also be carried and their arils removed by ants [64]. The size of the seeds and aril removal are key factors in the species’ seed germination [65,66].

**Fig 1:**
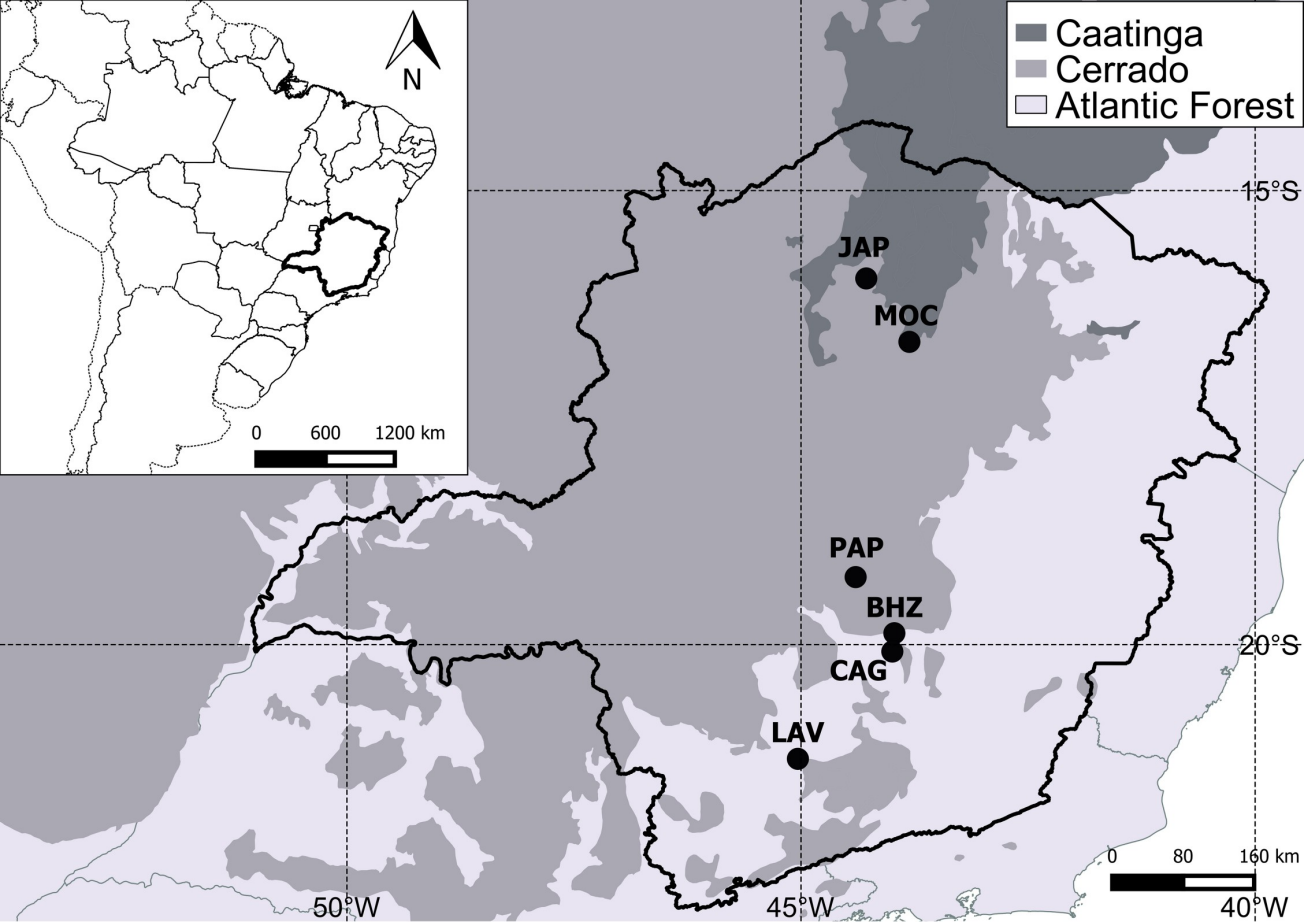
Location of the six populations *of Copaifera langsdorffii* selected for this study (codes in Table 1).

**Fig 2:**
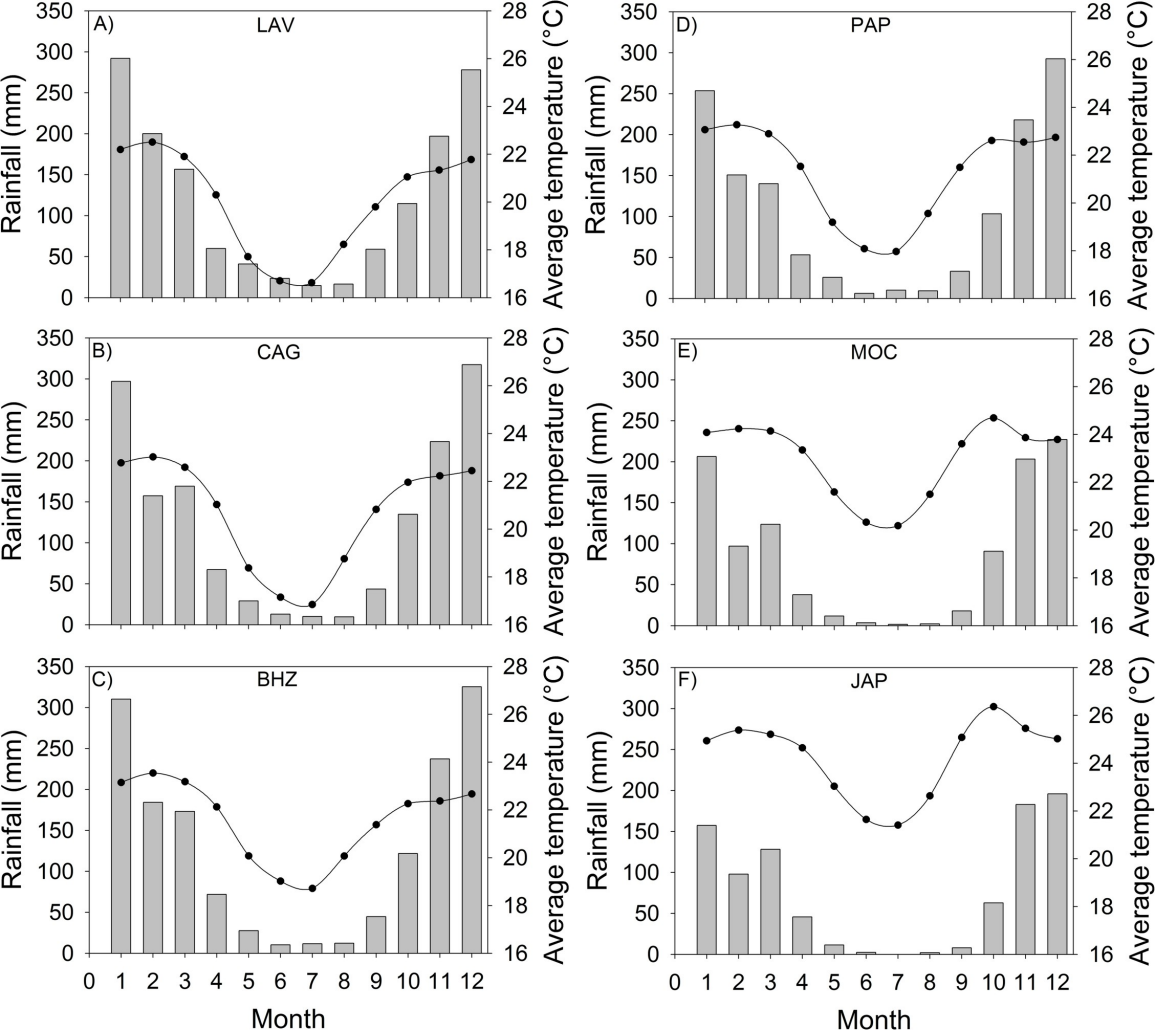
Month rainfall (mm) and month average temperature (^o^C) for the six sites selected for this study (codes in Table 1). Climatic data for the last 54 years (1961–2014) for each location were obtained from the Brazilian National Institute of Meteorology (INMET, 2015).

This study was conducted in six populations of *C*. *langsdorffii* in three biomes in the state of Minas Gerais in southeastern Brazil (Fig 1), distributed across a climatic gradient (Table 1, Fig 2). Phenological studies conducted in these populations of *C*. *langsdorffii* indicate differences temporal in vegetative phenology along the gradient, with a pronounced leaf shedding over the driest periods in populations of the most arid environments (unpublished data). The population named JAP occurs in a seasonally dry forest in the Caatinga biome. This forest formation presents trees with height that can exceed 25 meters and leaf abscission of species is higher than 90% in the dry season [67]. The soils are rich in nutrients and leaf fall contributes to soil fertility [68]. Population named MOC occurs in Cerrado *stricto sensu*, a savanna vegetation in deep, acidic and nutrient-poor soils [68]. PAP population occurs in Cerradão, a forest formation in the Cerrado biome that presents large trees with closed canopy. The soils are deep, slightly acidic with a medium content of organic matter coming from the fall of the leaves in the dry season [68,69]. GAG population occurs in ferruginous rock field, a vegetation in the Cerrado-Atlantic Forest biomes transition, on top of mountains at altitudes above 900 m. This predominantly herbaceous-shrub vegetation occurs in very shallow soil with high iron content [70]. BHZ and LAV populations occur in a semideciduous forest in the Atlantic Forest biome. This type of vegetation has large trees forming a continuous canopy; the soils are deep and poor in mineral nutrients [71].

**Table 1:** Environmental and climatic characterization of *Copaifera langsdorffii* populations. In brackets are the values of interannual variation in climatic variables (coefficients of variation, CV = SD mean^-1^, expressed as percentages). Data were obtained from the Brazilian National Meteorology Institute (www.inmet.gov.br) for the period 1961–2014.

Description	Pop. JAP	Pop. MOC	Pop. PAP	Pop. BHZ	Pop. CAG	Pop. LAV
Population	Japonvar	Montes Claros	Paraopeba	Belo Horizonte	Canga	Lavras
Coordinates	15º58’S 44º16’W	16^o^40’S 43^o^48’W	19°20’S 44°24’W	19°53'S 43°58'W	20°04’S 43°59’W	21º15'S 45º02'W
Altitude (m)	804	645	763	842	1423	948
Habitat	Seasonally dry forest	Cerrado *strictu sensu*	Cerradão	Semideciduous montana forest	Ferruginous rock field	Semideciduous montana forest
Rainfall	858.0 (30.7)	1029.4 (26.2)	1295.3 (22.2)	1500.4 (21.9)	1490.1 (21.4)	1511.5 (18.1)
Temperature	24.2 (3.0)	23.0 (2.5)	21.3 (2.6)	21.5 (2.8)	20.74 (4.0)	20.0 (3.1)
Evapotranspiration	109.1 (4.3)	101.9 (3.5)	89.6 (3.4)	72.0 (3.6)	100.4 (5.2)	83.4 (3.8)
Sunshine	2876.1 (9.5)	2667.1 (9.9)	2667.4 (6.3)	2502.6 (8.4)	2224.4 (7.4)	2466.7 (9.6)
AI	0.6 (33.1)	0.7 (26.7)	1.0 (22.8)	1.2 (22.1)	1.1 (22.5)	1.3 (19.6)

Rainfall = Annual rainfall (mm); Temperature = average annual temperature (°C); Evapotranspiration = potential evapotranspiration (mm month^-1^); Sunshine = Total annual hours of bright sunshine (h); AI = average annual aridity index.

### Climatic variables

Climatic data for the last 54 years (1961–2014) for each location (Table 1, Fig 2) were obtained from the Brazilian National Institute of Meteorology [72]. The meteorological stations are near the studied sites with maximum distance of about 20 km. To characterize the climatic heterogeneity of the environments, we calculated the interannual variability through the coefficient of variation (CV = SD mean^-1^, expressed as percentage) of each climatic variable. Populations located further north are under lower mean annual rainfall (858.0 and 1029.4 mm) and higher interannual rainfall variability (30.7 and 26.2%). Populations further south are under a higher mean annual rainfall (1490.1 and 1511.5 mm) and less interannual rainfall variability (21.4 and 18.1%). Northern areas also present higher annual temperature, higher annual hours of bright sunshine and higher evapotranspiration. From the climatic data, we calculated an aridity index (AI) monthly for each location using the formula: AI = P/PET, where P is total rainfall of the month and PET is monthly potential evapotranspiration at each location obtained from climatic stations [73]. Lower AI values correspond to more arid populations. For the analyses, we used the average annual aridity index (Table 1). A clear gradient in aridity was found, with populations further north subject to a more arid climate (AI = 0.6 and 0.7) and higher interannual variability (33.3 and 26.7%). Populations located further south are subject to lower aridity (AI = 1.1 and 1.3) and lower interannual variability (22.5 and 19.6%).

### Morphological and physiological metamer traits

Between April and May of 2013, 20 adult individuals of *C*. *langsdorffii* were selected at each population, except in the population named BHZ where only 12 individuals were sampled due to the relative inaccessibility of trees. From each individual, we collected a total of 22 metamers (i.e., internode, petiole and the corresponding leaf); 11 metamers exposed to the sun and 11 metamers in the shade were collected [74]. Metamers in the last nodes with mature and fully expanded leaves were sampled. Once collected, metamers were immediately photographed with a millimeter scale for subsequent determination of leaf area (LA in cm^2^), the length of the petiole and the length between nodes (PL and IL, respectively, in cm) using the Image J software. Metamers were put in paper bags and dried in an oven at 70° C for 72 h. Each part of the metamer was weighed separately to obtain the dry mass. We calculated specific leaf area (SLA; area of the leaf blade by dry mass unit, in cm^2^g^-1^), the metamer leaf area ratio (LARm; area of the leaf blade per dry mass unit of the metamer; in cm^2^g^-1^), the specific length of the petiole (SPL; length of the petiole per dry mass unit of the petiole; in cm g^-1^), and specific length of internode (SIL; internode length per dry mass unit of internode, in cm g^-1^) [52].

Chlorophyll fluorescence measurements were conducted on three individuals of *C*. *langsdorffii* from each population. In each individual, chlorophyll fluorescence was measured in 6 leaves, 3 exposed to sun and 3 shaded. The chlorophyll fluorescence measurements were performed at midday, using a portable fluorometer (PAM-2500, Walz Germany). The potential quantum yield of photosystem II was calculated by Fv/Fm = (Fm-F_0_)/Fm, where Fm and F_0_ are the fluorescence maximum and minimum, respectively. Fm and F_0_ were measured after 30 minutes of dark adaptation. Light saturation curves were obtained using the light curve program of the fluorometer, and were used to determine maximum apparent photosynthetic electron transport rate (ETRmax) and saturating photosynthetically active photon flux density (PPFDsat) [75]. In all populations, the chlorophyll fluorescence measurements were performed in non-overcast days (more than 10 hours of bright sunshine) with PPFD higher than 1500 μmolm^-2^s^-1^ and temperature between 27 and 30°C in April during the transition of the wet to dry season.

### Data analysis

In order to analyze the partition of the ITV in different hierarchical levels we performed generalized linear mixed models (GLMM) using functions implemented in the 'nlme' package [76] using the statistical software R [77]. Variance in morphological and physiological traits was partitioned across the following hierarchical levels: among populations, among individuals within populations, among leaves within individuals in different light conditions and leaves within individuals in the same light conditions. The final level was used as the error term [15,16]. F-tests for each metamer trait were conducted using the appropriate error terms, considering the variation among populations as a fixed effect and other explanatory variables as random effects [78].

To investigate association of morphological and physiological traits with specific climatic variables we performed multiple regression analyses using generalized linear models (GLM) for sun and shade metamers separately. These analyses included predictor variables characterizing the climate of population sites: average annual temperature, annual rainfall, evapotranspiration, annual hours of bright sunshine and average annual aridity index. After the initial model fit, a stepwise model selection routine was used to include only the variables that collectively resulted in the minimum value of the Akaike information criterion (AIC) [79]. For each trait (response variable), we used mean values of each individual. The models were compared using ANOVA.

Phenotypic plasticity was estimated as the percentage of change in the mean trait value for different light conditions (sun and shade metamers). The phenotypic plasticity of each individual (P_i_) was calculated as P_i_ = [(Xh—X_l_) / X_h_] * 100, where X_h_ is the highest average value and X_l_ is the lowest average value of a particular trait between the two light conditions [35]. The plasticity of each population (P) was calculated as the mean of P_i_ of all individuals of the population.

We tested the effect of climatic heterogeneity on phenotypic plasticity using multiple regression analyses through GLM. Interannual variation in average annual temperature, annual rainfall, evapotranspiration, total annual hours of bright sunshine and average annual aridity index were used as explanatory variables. After the initial model fit, a stepwise model selection routine was used to include only the variables that collectively resulted in the minimum value of the Akaike information criterion (AIC) [79]. For each trait (response variable), we used phenotypic plasticity values (P_i_) of all individuals of each population. We also performed a multiple regression analysis considering the average plasticity of all morphological traits (overall morphological plasticity) and another analysis considering the average plasticity of all physiological traits (overall physiological plasticity) as response variables.

Data set of morphological and physiological traits are in S1 and S2 Tables, respectively. Data were analyzed using the software R [77]. All models were built using the appropriate error distribution considering the nature of each response variable, followed by model criticism via residual analysis [78]. All models were compared with null models and the appropriateness of the models was tested by residual analysis [78].

## Results

### Partition of the intraspecific trait variation

GLMMs revealed that all morphological traits significantly varied across all the hierarchical levels considered (Table 2). For all morphological traits, the highest proportion of variance (41.5–68.4%) was found among metamers within individuals in the same light condition (error term) (Table 2). Significant variation among light conditions within individuals for all traits was found, ranging from 5.8 to 67.1%. The differences among individuals within populations for morphological traits varied from 3.9 to 29.3%. No variation among individuals within populations was detected for any of the physiological traits. Significant variation among populations was found for all traits (8.8 to 38.8%), with exception of Fv/Fm. High divergence among populations was found for leaf area (25.5%), SPL (33.7%), ETRmax (26.1%), and PPFDsat (38.8%) (Table 2).

**Table 2:** Hierarchical partitioning of variance (in percentage) for morphological and physiological traits of metamers in *Copaifera langsdorffii*. Variance components and significance levels were determined with GLMM.

		Level		
*Morphological* *Traits*	Population	Plant [Population]	Leaf different light [Plant, Population]	Leaf same light [Error]
Leaf area	25.5***	20.1***	12.9***	41.5
Petiole length	15.8***	29.3***	9.4***	45.4
Internode length	12.5***	24.5***	8.0***	54.9
SLA	11.9***	5.1***	17.9***	65.1
LARm	8.8***	3.9***	20.9***	66.4
SPL	33.7***	13.6***	5.8***	46.8
SIL	10.6***	10.6***	10.4***	68.4
Average	17.0	17.2	10.7	53.7
*Physiological* *Traits*				
ETRmax	26.1**	5.1^−7^ NS	48.4***	25.5
PPFDsat	38.8***	5.9^−5^ NS	16.0*	45.1
Fv/Fm	7.8^-8^NS	2.0^−11^ NS	67.1***	32.9
Average	21.6	2.0^−5^	43.9	34.5

***P < 0.001

**P < 0.01

*P < 0.05

NS *P* > 0.05 in GLMM

### Association of metamer traits with climatic variables

**Fig 3:**
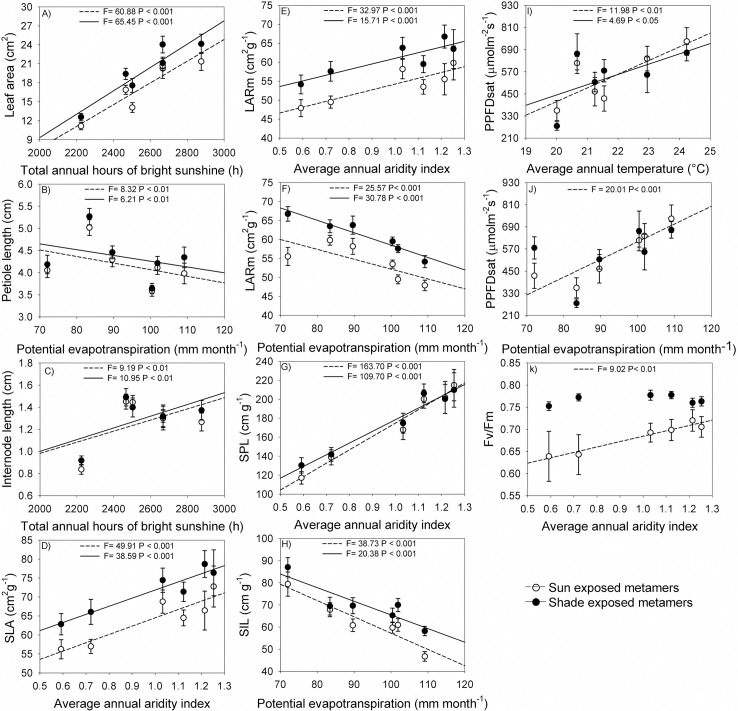
Relationship between different morphological and physiological traits of metamers and climatic variables for populations of *Copaifera langsdorffii* for: A) leaf area (cm^2^); B) petiole length (cm); C) internode length (cm); D) SLA, specific leaf area (cm^2^ g^-1^); E) and F) LARm, leaf area ratio of the metamer (cm^2^ g^-1^); G) SPL, specific petiole length (cm g-^1^); H) SIL, specific internode length (cm g-^1^); I) and J) PPFDsat, saturating photosynthetically active photon flux density (mmolm^-2^s^-1^); K) Fv/Fm, potential quantum yield of photosystem II. Average values and SE of each population are shown (for morphological traits *N* = 112 and physiological traits *N* = 18).

Morphological and physiological traits of metamers, except ETRmax, were significantly (*P* < 0.05) associated with climatic variables (Table 3, Fig 3). Aridity explained the variation for four of the ten metamer traits analyzed in this study (Table 3, Fig 3). Populations in more arid climate have metamers with lower SLA (sun *R^2^* = 0.30, *P* < 0.001 and shade *R^2^* = 0.25, *P* < 0.001, Fig 3D), lower leaf area per metamer mass (LARm; sun *R^2^* = 0.22, *P* < 0.001 and shade *R^2^* = 0.17, *P* < 0.001, Fig 3E), lower specific length of the petiole (SPL; sun *R^2^* = 0.59, *P* < 0.001 and shade *R^2^* = 0.49, *P* < 0.001, Fig 3G) and lower Fv/Fm in sun-exposed metamers (*R^2^* = 0.32, *P* < 0.01, Fig 3K). Although the Fv/Fm showed significant differences along the aridity gradient in sun-exposed metamers, the differences among populations in extremes of aridity gradient were low, not more than 10% (Fig 3K). Compared to sun-exposed leaves, the Fv/Fm values in shade leaves were higher, however they were not influenced by the climatic variables along the gradient analyzed. The differences among sun and shade leaves along of the climatic gradient for Fv/Fm can explain the fact that no significant differences among populations were found for this trait when the data regarding sun and shade leaves were grouped in the partitioning of variance analysis (Table 2).

**Table 3:** Results of multiple regression analyses of morphological and physiological traits of metamers of *Copaifera langsdorffii* with climatic variables. For each trait, mean values of each individual were used.

Regression		AIC	*R*^2^	*F*	*P*			AIC	*R*^2^	*F*	*P*
	*Metamers in sun*						*Metamers in shade*				
*Morphological traits*						*Morphological traits*					
Leaf area = -25.90+0.02Sunshine		673.51	0.35	60.88	<0.001	Leaf area = -27.25+0.02Sunshine		683.60	0.37	65.43	<0.001
Petiole length = 6.06–0.02Evap		285.13	0.06	8.32	<0.01	Petiole length = 6.08–0.02Evap		297.05	0.05	6.21	<0.01
Internode length = -0.10+5.26E-04Sunshine		107.81	0.07	9.19	<0.01	Internode length = -0.11+5.44E-04Sunshine		95.68	0.08	10.95	<0.01
SLA = 42.05+22.68AI		802.86	0.30	48.91	<0.001	SLA = 50.91+20.77AI		809.65	0.25	38.59	<0.001
LARm = 50.41+12.34AI+0.09Evap		766.46	0.22	16.84	<0.001	LARm = 81.30+4.16AI-0.26Evap		773.20	0.21	15.71	<0.001
SPL = 32.83+142.41AI		1079.10	0.59	163.70	<0.001	SPL = 54.15+124.78AI		1094.30	0.49	109.70	<0.001
SIL = 128.94–0.72Evap		915.10	0.26	38.73	<0.001	SIL = 121.93–0.57Evap		933.54	0.15	20.38	<0.001
*Physiological traits*						*Physiological traits*					
ETRmax = 46.17		156.00	1.81^−3^	0.03	0.87	ETRmax = 59.51		169.98	1.47^−3^	0.02	0.88
PPFDsat = -900.05+35.80Temp+7.15Evap		224.73	0.57	12.12	<0.001	PPFDsat = -671.56+55.79Temp		236.57	0.18	4.69	<0.05
Fv/Fm = 0.56–0.12AI		-58.59	0.32	9.02	<0.01	Fv/Fm = 0.77		-95.75	0.02	0.42	0.53

Rainfall = Annual rainfall (mm); Temp = Average annual temperature (°C); Evap = Potential evapotranspiration (mm month ^-1^); Sunshine = Total annual hours of bright sunshine (h); AI = Average annual aridity index. For morphological traits *N* = 112 and physiological traits *N* = 18.

Evapotranspiration explained the variation in four traits, with populations located in areas with higher evapotranspiration having lower petiole length (sun *R^2^* = 0.06, *P* < 0.01 and shade *R^2^* = 0.05, *P* < 0.01, Fig 3B), lower specific internode length (SIL, sun *R^2^* = 0.126, *P* < 0.001 and shade *R^2^* = 0.15, *P* < 0.001, Fig 3H), lower LARm in shade exposed metamers (sun *R^2^* = 0.18, *P* < 0.001 and shade *R^2^* = 0.21, *P* < 0.001,Fig 3F) and higher PPFDsat in sun exposed metamers (*R^2^* = 0.53, *P* < 0.001, Fig 3I). In turn, annual hours of bright sunshine was the variable which better explained variation in other two leaf morphological traits, leaf area (sun *R^2^* = 0.35, *P* < 0.001 and shade *R^2^* = 0.37, *P* < 0.001, Fig 3A) and internode length (sun *R^2^* = 0.07, *P* < 0.001 and shade *R^2^* = 0.08, *P* < 0.001, Fig 3C). Populations in sites with higher annual hours of bright sunshine exhibited higher leaf area and longer internode. Finally, the temperature positively affected PPDFsat (sun *R^2^* = 0.39, *P* < 0.01 and shade *R^2^* = 0.18, *P* < 0.05, Fig 3I).

### Phenotypic plasticity and its association with climate heterogeneity

Physiological traits showed higher phenotypic plasticity than morphological traits, with the overall plasticity ranging from 17.6 to 31.0% and 12.0 to 15.3%, respectively (S3 Table). Phenotypic plasticity also varied among populations mainly for physiological metamer traits. Populations showed a latitudinal gradient in phenotypic plasticity, with northern populations having higher plasticity than southern populations (S3 Table).

**Fig 4:**
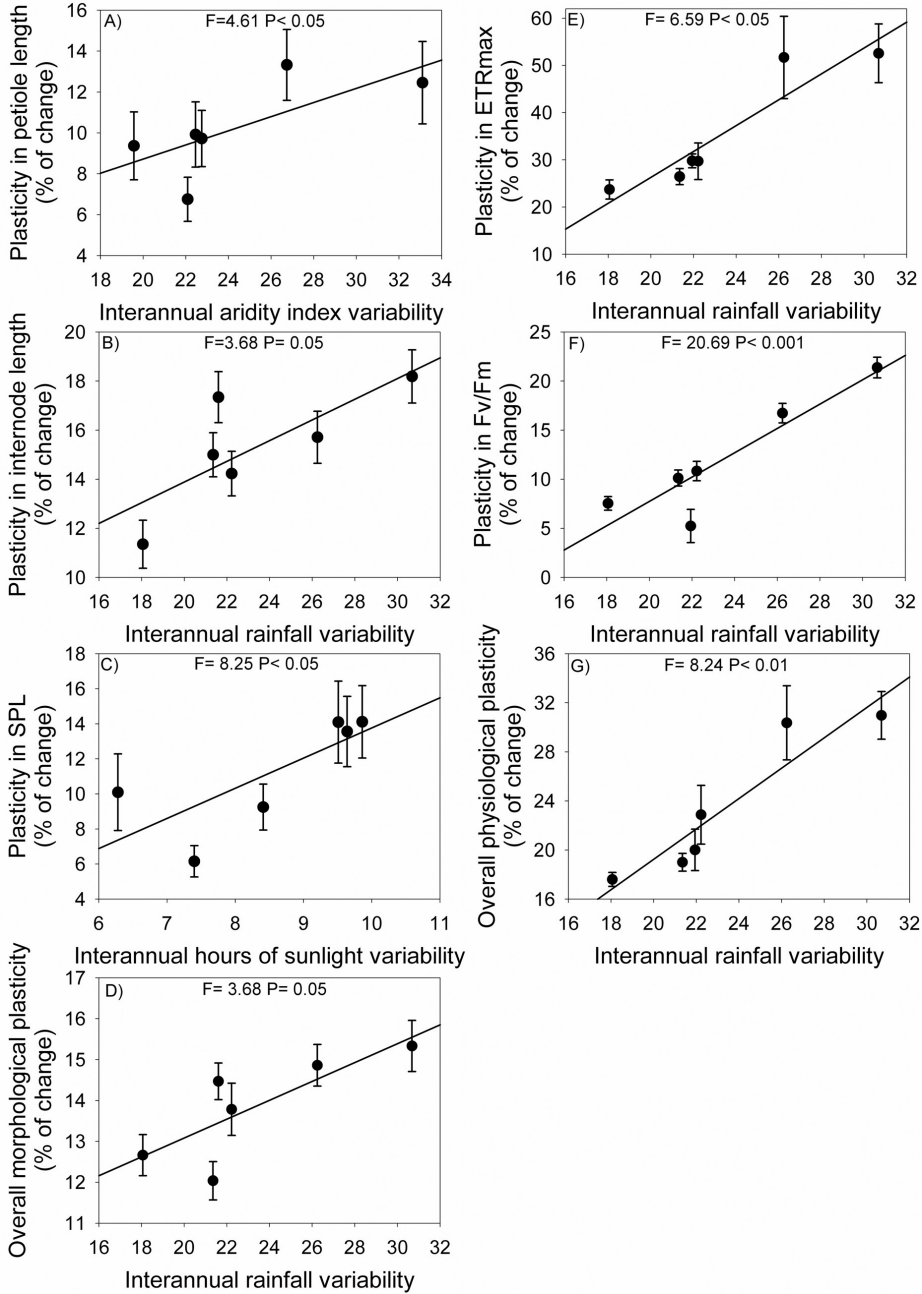
Relationship between trait phenotypic plasticity and interannual variation in climatic variables in populations of *Copaifera langsdorffii* for: A) petiole length; B) internode length; C) SPL, specific petiole length; D) overall morphological plasticity (measured as the arithmetic mean of the percentage of change for all morphological traits); E) ETRmax, maximal electron transport rate; F) Fv/Fm, potential quantum yield of photosystem II; G) overall physiological plasticity (measured as the arithmetic mean of the percentage of change for all physiological traits). Average values and SE of each population are shown (for morphological traits *N* = 112 and physiological traits *N* = 18).

Multiple regressions showed that phenotypic plasticity of metamer traits was positively associated mainly with interannual variation (heterogeneity) in rainfall (Table 4). The overall plasticity of both morphological and physiological traits and plasticity of internode length, ETRmax and Fv/Fm were positively associated with interannual variation in rainfall (Table 4, Fig 4). When compared with morphological traits, phenotypic plasticity of physiological traits was more associated with rainfall heterogeneity (*R^2^* = 0.06, *P* < 0.06, and *R^2^* = 0.30, *P* < 0.01, respectively). In addition, plasticity in petiole length was positively associated with interannual variation in aridity index and plasticity in SPL with variation in annual hours of bright sunshine.

**Table 4:** Results of multiple regression analyses of phenotypic plasticity of morphological and physiological traits of metamers of *Copaifera langsdorffii* with interannual variation in climatic variables (coefficients of variation, CV = SD mean^-1^).

Regression	AIC	*R*^2^	*F*	*P*
*(A) Morphological traits*				
Leaf area = 0.16	-155.31	8.40^−3^	0.93	0.34
Petiole length = 0.03 + 0.32AI	-264.37	0.03	4.61	<0.05
Internode length = 0.15 + 0.45Rainfall	-185.23	0.02	3.68	0.06
SLA = 0.13	-231.01	0.02	3.28	0.08
LARm = 0.13	-218.01	0.01	2.61	0.11
SPL = -0.03 + 1.71Sunshine	-229.07	0.06	8.25	<0.01
SIL = 0.18	-161.41	2.40^−3^	0.25	0.62
Overall plasticity = 0.08 + 0.25Rainfall	-321.43	0.03	3.68	0.06
*(B) Physiological traits*				
ETRmax = 0.29 + 2.74Rainfall	-6.28	0.25	6.59	<0.01
PPDFsat = 0.23	-18.95	1.30^−4^	2.10^−3^	0.96
Fv/Fm = -0.17 + 1.24Rainfall	-55.37	0.54	20.60	<0.001
Overall plasticity = -0.26 + 0.07Rainfall	-39.93	0.30	8.24	<0.01

Rainfall = Annual rainfall (mm); Sunshine = Total annual hours of bright sunshine (h); AI = Average annual aridity index. For morphological traits *N* = 112 and physiological traits *N* = 18.

## Discussion

According to our hypothesis, *Copaifera langsdorffii* shows high intraspecific trait variation across the climatic gradient. For most of the analyzed traits, variation among the populations is explained mainly by aridity index or evapotranspiration. Moreover, the overall phenotypic plasticity evaluated through the comparison between metamers exposed to sun and shade, is associated with the interannual variation in rainfall. Northern populations that are subject to lower annual rainfall and higher interannual variation in rainfall have greater phenotypic plasticity.

Our results pointed that the largest fraction of total morpho-physiological trait variation is found within individuals. The total variance within individuals for the morphological and physiological traits, including the variance among metamers both in different and in similar light conditions, was on average 64.4% and 78.4%, respectively. These results are in accordance with other studies which have demonstrated greater variation within individuals [14–16,25,80]. Physiological variation among metamers of the same plant exposed to different light conditions (sun and shade) was fourfold that of morphological traits, consistent with the higher plasticity found for physiological traits. Higher phenotypic plasticity in physiological traits when compared to morphological traits has also been described for other two tree species of the Cerrado and Atlantic Forest [37]. Large variation among sun and shade metamers within individuals is important for maximizing photosynthesis through the optimization of light capture across tree crown. Also, high phenotypic plasticity has been considered important for allowing plants to successfully respond to changing environmental conditions [12,55].

High trait variation was found among populations of *C*. *langsdorffii* for morphological and physiological traits (8.8 to 38.8%) in comparison with other tree species [14,15]. This variation among populations may be the result of natural selection leading to the development of morphological and physiological adaptations to local environments [26,36]. Thus, genetic differentiation among *C*. *langsdorffii* populations can explain part of the phenotypic divergence among them, with genotypes adapted to local environmental conditions. Our experimental design did not account to determine the genetic differentiation among populations for the traits, i.e., its local adaptation. However, our study allowed to evaluate the degree of phenotypic plasticity of the populations, which was high for the most of the traits, and thus it can explain part of the observed variation among the populations. To estimate the relative contribution of genetic variation and phenotypic plasticity to phenotypic variation of the metamer traits, additional studies evaluating progenies in common garden experiments should be performed.

*C*. *langsdorffii* populations from more xeric habitats had low values of SLA and LARm, which can to lead a reduction in water loss by transpiration, enhancing water use efficiency [12]. Several studies analyzing the relationship between climate and leaf morphological traits in several ecosystems around the world have found patterns similar to ours [16,21,52,54,79,81–83]. Plants in arid environments tend to have lower LARm, suggesting that this trait is associated with low water availability [52,84]. Individuals from sites with higher hours of bright sunshine (JAP, MOC and PAP) had higher leaf area. Larger leaves require more hydraulic and biomechanical support, which can be produced by low SPL and SPI [85], increasing efficiency of biomass investment for foraging [52]. Other environmental factors such as light heterogeneity are also important to determine morphological, anatomical, hydraulic and architectural characteristics of the leaf petiole [86–88], influencing leaf photosynthetic capacity [89].

Sun-exposed metamers from plants located in more xeric climate also had lower values of Fv/Fm. This reduction of the quantum yield of photosynthesis indicates higher damage on photosystem II by excessive light (photoinhibition) [10,90] in sun-exposed leaves in plants of arid environments. Populations from sites that have higher evapotranspiration had higher values of saturating photosynthetically active photon flux density (PPFDsat). Leaves exposed to sunlight from populations of more arid climates (JAP and MOC) needed approximately twice as much light to saturate photosynthesis compared to those from less arid climates. This difference in light requirement to saturate photosynthesis could be interpreted as a response to the sunnier environments of the more arid climates, in spite of a certain degree of photoinhibition. A high incidence of light combined with water stress can compromise the photosynthetic apparatus of plants leading to photoinhibition even in drought-adapted species with xeromorphic traits [10].

Plants of sites with higher hours of bright sunshine (Jap, Moc and Par) had large leaf area. Such sites have lower annual rainfall when compared with the other areas. Several studies have shown that plants in sites of low rainfall tend to reduce their leaf area [16,54,91]. However a recent study [53] showed that leaf size is regulated by a complex network of environmental variables, and that sites with annual rainfall greater than 750 mm, temperature and irradiance are the most important variables affecting positively the size of the leaves. In our study, the annual rainfall ranges from about 850–1500 mm. In this way, the positive effect of hours of bright sunshine in leaf area of *C*. *langsdorffii* corroborates this prediction.

Overall, we found greater phenotypic plasticity for the overall morphological and physiological metamer traits in populations from habitats with greater interannual variation in rainfall, which correspond to drier habitats. Our results are in accordance with studies reporting a positive association between phenotypic plasticity and annual variability of rainfall [12,57]. These results support the theoretical predictions of greater plasticity in more heterogeneous environments [33,48,49]. *C*. *langsdorffii* populations from locations with greater environmental heterogeneity had greater plasticity in petiole length, internode length, SPL, ETRmax, Fv/Fm and also considering the overall data for both morphological and physiological traits. We evaluated phenotypic plasticity by comparing traits between sun-exposed and shade metamers. Sun-exposed metamers compared to those from shade portion of the crown, are subjected to conditions of greater water stress due to high irradiance, higher temperatures, and wind action on the outermost portion of the canopy [46,92,93].

The higher phenotypic plasticity in *C*. *langsdorffii* populations from drier habitats and with greater interannual variation in rainfall may be a result from higher differences in the stressful conditions of metamers in relation to that experienced by populations from more mesic habitats, which presented less climatic heterogeneity. The metamer traits, petiole length, internode length and SPL are directly related to the hydraulic and biomechanical aspects of the leaves [56,85,94,95]. Shorter internode and petiole reduce the resistance to water flow and lower SPL values increase the content of conducting vessels per unit of length [96–98], resulting in an increase of water supply in the leaf blade [97,99]. In more stressful environments it also expected higher differences in photosynthetic traits between shade and sun-exposed leaves justifying the highest plasticity of Fv/Fm and ETR. It should be noted that *C*. *langsdorffii* is a long-lived and deciduous tree that cope with several environmental conditions during its life and has leaf fall every year in dry season. So, the greater plasticity in heterogeneous environments allows that the new metamers formed each year present traits linked to the hydraulic of the leaves and photosynthesis partly shaped by climatic conditions of the corresponding year, contributing for the persistence of the populations. Although it has not been evaluated in this study, some authors have demonstrated that this plasticity can be adaptive in some cases [12,100], i.e, plants with more ability to change its phenotype according to environmental conditions should selected in more heterogeneous environments.

In summary, our results demonstrate a high intraspecific metamer trait variation in *C*. *langsdorffii* across a climate gradient. The trait variation among populations is shaped mainly by aridity and evapotranspiration. A considerable part of this variation is due to phenotypic plasticity. The wide variation in metamer traits found in *C*. *langsdorffii* coupled with its ability to modify these traits in response to different climate conditions can explain the success of the species over a range of different habitats across its wide geographic distribution in the Cerrado, Atlantic forest and Caatinga. Populations from environments with greater interannual climatic heterogeneity could be better suited to cope with future climate changes because of their xerophytic features and their higher levels of phenotypic plasticity.

## Supporting information

S1 Table Data set of morphological traits of metamers in *Copaifera langsdorffii*. (XLSX)

S2 Table Data set of physiological traits of metamers in *Copaifera langsdorffii*. (XLSX)

S3 Table Mean values of phenotypic plasticity (measured as percentage of change) for different metamer traits in populations of *Copaifera langsdorffii*. In brackets are the values of standard error.
(DOCX)
