# Supplementary material for: Climatic factors shaping intraspecific leaf trait variation of a neotropical tree along a rainfall gradient
Source: PLoS One. 2018 Dec 6;13(12):e0208512. doi: 10.1371/journal.pone.0208512 (PMC6283565; doi:10.1371/journal.pone.0208512)
Supplement: S3 Table — In brackets are the values of standard error. (DOCX) [file pone.0208512.s003.docx]

**Supporting information**

**S3 Table: Mean values of phenotypic plasticity (measured as percentage of change) for different metamer traits in populations of *Copaifera langsdorffii*.** In brackets are the values of standard error.

| **Traits** |  |  | **Populations** |  |  |  |
| --- | --- | --- | --- | --- | --- | --- |
| *Morphological traits* | JAP | MOC | PAP | BHZ | CAG | LAV |
| Leaf area | 16.5 (2.5) | 16.6 (3.2) | 16.2 (2.8) | 19.1 (3.5) | 13.8 (2.6) | 15.7 (1.7) |
| Petiole length | 12.5 (2.0) | 13.3 (1.7) | 9.7 (1.4) | 6.8 (1.1) | 9.9 (1.6) | 9.4 (1.7) |
| Internode length | 18.2 (2.6) | 15.7 (2.5) | 14.2 (2.1) | 17.3 (2.5) | 15.0 (2.1) | 11.4 (2.3) |
| SLA | 12.1 (2.1) | 13.8 (1.7) | 14.7 (2.3) | 15.8 (2.0) | 10.8 (1.6) | 9.8 (1.5) |
| LARm | 13.1 (2.1) | 13.9 (1.7) | 14.5 (2.5) | 17.1 (2.3) | 10.9 (1.7) | 10.2 (1.6) |
| SPL | 14.1 (2.3) | 14.1 (2.1) | 10.1 (2.2) | 9.3 (1.3) | 6.2 (0.9) | 13.6 (2.0) |
| SIL | 21.0 (2.8) | 16.5 (2.6) | 17.1 (2.6) | 15.9 (2.6) | 17.7 (2.4) | 18.8 (2.8) |
| Overall plasticity | 15.3 (1.5) | 14.9 (1.2) | 13.8 (1.5) | 14.5 (1.1) | 12.0 (1.1) | 12.7 (1.2) |
| *Physiological traits* |  |  |  |  |  |  |
| ETRmax | 52.6 (15.2) | 51.7 (21.4) | 29.7 (9.5) | 29.8 (3.6) | 26.4 (4.2) | 23.7 (5.0) |
| PPFDsat | 19.0 (4.5) | 22.7 (10.0) | 28.1 (8.1) | 25.0 (12.5) | 20.4 (9.2) | 21.6 (6.1) |
| Fv/Fm | 21.4 (2.6) | 16.7 (2.4) | 10.8 (2.4) | 5.2 (4.1) | 10.1 (2.0) | 7.5 (1.7) |
| Overall plasticity | 31.0 (4.8) | 30.4 (7.4) | 22.9 (5.9) | 20.0 (4.1) | 19.0 (1.8) | 17.6 (1.4) |
